# “Democratizing” artificial intelligence in medicine and healthcare: Mapping the uses of an elusive term

**DOI:** 10.3389/fgene.2022.902542

**Published:** 2022-08-15

**Authors:** Giovanni Rubeis, Keerthi Dubbala, Ingrid Metzler

**Affiliations:** Karl Landsteiner University of Health Sciences, Krems an der Donau, Austria

**Keywords:** artificial intelligence, big data, ethics, digital technologies, democratization

## Abstract

**Introduction:** “Democratizing” artificial intelligence (AI) in medicine and healthcare is a vague term that encompasses various meanings, issues, and visions. This article maps the ways this term is used in discourses on AI in medicine and healthcare and uses this map for a normative reflection on how to direct AI in medicine and healthcare towards desirable futures.

**Methods:** We searched peer-reviewed articles from Scopus, Google Scholar, and PubMed along with grey literature using search terms “democrat*”, “artificial intelligence” and “machine learning”. We approached both as documents and analyzed them qualitatively, asking: What is the object of democratization? What should be democratized, and why? Who is the demos who is said to benefit from democratization? And what kind of theories of democracy are (tacitly) tied to specific uses of the term?

**Results:** We identified four clusters of visions of democratizing AI in healthcare and medicine: 1) democratizing medicine and healthcare through AI, 2) multiplying the producers and users of AI, 3) enabling access to and oversight of data, and 4) making AI an object of democratic governance.

**Discussion:** The envisioned democratization in most visions mainly focuses on patients as consumers and relies on or limits itself to free market-solutions. Democratization in this context requires defining and envisioning a set of social goods, and deliberative processes and modes of participation to ensure that those affected by AI in healthcare have a say on its development and use.

## 1 Introduction

In his seminal work What Tech calls Thinking: An Inquiry into the Intellectual Bedrock of Silicon Valley, Adrian Daub (2020) describes the way Big Tech uses and reframes concepts like “disruption” and “communication” to shape our understanding of the goals and purposes of the industry. Daub argues that by reframing these concepts, narratives are implanted into the collective consciousness that explain and legitimize the way digital companies aim to change the world. In his view, digital technologies, especially Big Data applications and machine learning software, often referred to as Artificial Intelligence (AI), are not mere tools for improving communication and data exchange, optimizing work processes, or enabling commodification of social goods. Rather, these tools are framed as enablers of a new, and of course better, way of life. Big Tech is more than just selling products or services; it is about making the world a better place (Daub, 2020).

We are witnessing a similar tendency in discourses on digital technologies and AI in medicine and healthcare. Digital technologies are developed and used for a wide variety of diagnostic and therapeutic practices such as health data management, image recognition, decision support systems and assistive technologies (Briganti and Le Moine, 2020; Mishra, 2022). Value-laden terms like “disruption” (Rubeis, 2020) and “revolution” (Topol, 2012) are prominent in the discourse on these technologies. Recently, the term “democratization” has been added to this list. According to Eric Topol, one of the most prominent voices in the discourse on AI in medicine and healthcare, these technologies will transform medical practices and structures of healthcare systems and thus democratize medicine (Topol, 2012; Topol, 2015; Steinhubl and Topol, 2018; Topol, 2019). In this view, AI is more than just a new tool for improving isolated medical practices. Following Topol, the ultimate goal is “deep empathy” (Topol, 2019). Deep empathy refers to the optimization of data use and work processes, which will free physicians from time-consuming and mechanical tasks, thus leaving them more room to focus on their relationship with patients (Topol, 2019). Topol describes deep empathy as the culmination of a process that combines digitalization and democratization. Another crucial aspect in this discussion is the ownership of, control over, and access to personal health data by patients. Topol links the “suppressive force of doctors to retain control of patient data” to paternalism (Topol, 2019, p.270) and claims that “medical paternalism would fade as consumers didn’t simply generate their information but owned it” (Topol, 2019, p.24). In this view, the patient-as-consumer and data-owner is empowered and can face healthcare professionals on an equal level. This “deep medicine”, as termed by Topol, is enabled by AI-technologies and the use of big data. When the optimization of workflow of healthcare professionals and the empowerment of patients converge, we will get rid of paternalism for good and thus democratize medicine.

However, this particular use of the term democratization is not universally shared within the ongoing debate on AI in medicine and healthcare. “Democratizing AI” is a vague term that encompasses various meanings, issues, and visions. Its use extends in nuances within two poles, each reflecting competing understandings of the power of biomedical technologies and their agency in innovation processes (Timmermans and Berg, 2003; Metzler and Åm, 2022). One pole consists of the framing of AI as a transformative agent that can democratize medicine and healthcare. Medicine and healthcare are the objects that ought to be made more democratic, and data-intensive technologies and AI are the means to achieve this goal. The other pole consists of uses in which AI is the object that ought to be democratized. This vision is articulated in various nuances. Some actors underline a need to democratize access to technical tools that help develop AI. The tools include open access to code libraries, developer tools, and data sets (Garvey, 2018; Bhattacharya et al., 2021),collaborative learning and crowdsourcing (Bond et al., 2019a; Bond et al., 2019b; Lyu et al., 2020), accessible interfaces (Vanhorn and Çobanoğlu, 2021) and end-user machine learning systems (Traub et al., 2019). Access to these tools allows biomedical experts without software development skills to contribute to wider use of AI in healthcare. There are also calls to “democratize” AI algorithms by preventing sampling bias and tackling the under-representation of groups in training data (Mulvenna et al., 2021; Wong, 2019). Last but not the least, some actors also call for making the development and use of AI-based technologies an object of democratic governance (Nemitz, 2018; Himmelreich, 2022).

In this article, we map the different ways “democratizing AI” and the “democratization” of AI has been used in discourse on AI in medicine and healthcare and use the mapping for a normative discussion of the term. We begin by describing our methods. We then present and discuss our results. In the discussion section, we contextualize the different uses of the term democratizing AI with current approaches in medical and AI ethics. Since we address the topic of democratization in the context of medical AI from the perspective of normative ethics, our discussion will be a normative one. In the concluding section, we summarize the outcomes of our analysis.

## 2 Methods

This article is based on an empirical engagement with uses of the terms “democratization of AI” and “democratizing AI” in the discourse on medicine and healthcare.

### 2.1 Materials

In terms of materials, we used peer reviewed articles and grey literature. We searched for peer-reviewed articles on artificial intelligence within and outside healthcare using search terms “democrat*” and “artificial intelligence” or “machine learning”. We searched Scopus and PubMed databases through Ebsco search engine along with Nature, Science and Lancet journal databases and Google Scholar. We limited the search to English language but did not specify date range. The search resulted in 2071 articles. After deduplicating, we selected the articles that discussed AI and democratization in detail, as opposed to those that just mentioned the terms. We included all articles that refer to AI-based technologies without defining the term ourselves. This ensured an openness towards different interpretations of AI within the medical context. We complemented this material with documents from professional societies and international organizations that used some variation of the term “democratizing AI”. Finally, we included 35articles for analysis.

### 2.2 Conceptual lenses

We approached the literature as documents, in which the authors articulated understandings of the meaning and significance of “democratizing AI” in medicine and healthcare, often drawing on tacit understandings of the agency and power of AI and implicit theories on democracy. The documents we analyzed were of recent date (i.e., almost all of them were published after 2016 and several of them were published within the last 2 years) and diverse. They ranged from Editorials, over research articles, to guiding documents of professional societies. A cross-cutting theme was the literature’s future-oriented nature. Many articles discussed emerging trends, expected future developments, or called for actions to be taken. In light of the future-oriented nature of the discourse, we approached uses of the term “democratization” and “democratizing” AI as articulations of (sociotechnical) “visions” (Hilgartner, 2015; Jasanoff, 2015), i.e., understandings of the nature of desirable futures attainable through AI, and of the ways in which these futures can, or ought to, be achieved.

We analyzed the documents with “agnostic” lenses (Laurent, 2017). We did not select one definition of democratization as a normative baseline to critically assess our material, but strived to induce various definitions of “democratization” from the authors’ writings. This approach was informed by the conceptual understanding that, paraphrasing Tamar Sharon’s work on “common goods”, “a plurality of conceptualizations” (Sharon, 2018) of democratization and democracy are at work in the discourse on AI. Indeed, democracy can be understood as an “essentially contested concept” (Gallie, 1955). Most people agree on the value and importance of democracy, while they disagree on what democracy is, or ought to be. They agree that democracies are a desirable good, but they disagree on which actions ought to be taken to achieve this good in practice. In broadest terms, democracy refers to the “rule” (as of -cracy) of the “people” (demos) and denotes expectations on equality. However, there are disagreements on the range of objects that ought to be subjected to the rule of the people, on the desirable practices and institutions to organize that rule, and on the boundaries of the demos or people. Thus, definitions of democracies are also visions of what they ought to be. Similarly, “democratization” is a morally charged term. It problematizes a phenomenon as insufficiently democratic, while simultaneously giving moral power and legitimacy to the agents and means of democratization.

### 2.3 Data analysis

We analyzed the documents qualitatively to develop a better understanding of the uses of “democratization” in medicine and healthcare. We mapped four clusters of visions of democratizing AI from the materials, using the following questions to code the documents and distill and categorize clusters of visions:

• What is the object of democratization? What is the object that should be democratized, and why?
• Who is the “demos” (Doubleday and Wynne, 2011) who is said to benefit from, or that ought to be involved in, “democratizing AI?”
• What kind of theory of democracy is (often tacitly) tied to specific visions?

We phrased these questions after a first reading of the documents and engagements with scholarly literature on interactions between biomedical technologies and social orders in democratic societies (Timmermans and Berg, 2003; Marres, 2007; Doubleday and Wynne, 2011; Jasanoff, 2013). We then commenced with an intense analysis of a small random sample of documents (within the selected documents), seeking to maximize variations within this sample (Silverman, 2015). We coded them along the three questions, and distilled clusters of visions from this initial analysis.

When clustering visions of democratizing AI, we explored whether a specific vision was sufficiently different to be categorized as a distinct one or whether it could to be subsumed under a vision already deduced, following strategies of qualitative content analysis (Schreier, 2012). We analyzed the documents separately and discussed clusters of visions. Once we had agreed on the set of clusters, we used the remaining documents to validate the clusters.

## 3 Results

We identified four clusters of visions of democratizing AI within the analyzed material. In the following, we will outline these clusters.

### 3.1 Artificial intelligence for the people: Democratizing medicine and healthcare through Artificial intelligence

The first vision of’ “democratization” we identified is democratizing medicine and healthcare through AI. Following this view, democratization is based on two factors: data and the technologies to obtain and process them. Data include individual health data that may range from test results deposited in electronic health records, to behavioral data, or social media entries (Steinhubl and Topol, 2018; Topol, 2019; Weissglass, 2021). Data technologies encompass software like machine learning algorithms, data mining tools, and cloud computing, but also hardware like mobile devices (Mulvenna et al., 2021; Topol, 2019; Burnside et al., 2020; Weissglass, 2021). Mobile devices like smart phones or tablets allow users to generate and collect data outside of clinical settings or medical expertise. They are seen as the crucial device of democratizing healthcare, enabling clinicians to obtain real world data, e.g., through a digitally enhanced experience sampling method or ecological momentary assessment (Mulvenna et al., 2021). These methods are especially relevant for collecting behavioral and lifestyle data. Thus, data can be used to personalize treatment. Also, the fact that patients generate and collect this data themselves is seen as a democratizing effect (Steinhubl and Topol, 2018). Using mobile devices for data collection may also reduce access barriers to healthcare services (Weissglass, 2021). Digital technologies can help improve health surveillance by generating more and potentially better data, especially in settings with low healthcare coverage. In low-and middle-income countries (LMICs), this could contribute to better access to healthcare services and hence more democratic healthcare systems (Weissglass, 2021).

The connection between AI-technologies and health equity in terms of access is considered crucial for healthcare. One example discussed in the body of literature is a machine learning-based point-of-care screening tool for genetic syndromes in children (Porras et al., 2021). This deep phenotyping technology tool uses deep neural networks and facial statistical shape models to assess the risk of a child having one of the genetic syndromes covered by the technology. The tool, the authors argue, can identify the need of patients for referral to a specialist. It may thus assist physicians in their diagnostic practices, especially in areas where access to specialized care and genetic resources is scarce. Although the tool is no substitute to genetic diagnostics, it is referred to as a contribution to democratizing access to the healthcare resources needed (Porras et al., 2021).

Thus, in this first vision of democratizing AI, AI is imagined as a transformative agent that promises to democratize medicine and healthcare. The demos of this vision consists of individual citizens, often referred to as patients or consumers and mostly located in high-income countries (HICs), or a patient population mostly located in LMICs, who could benefit from the transformative power of AI-based technologies in redefining healthcare or providing healthcare through new means. The democratization of medicine and healthcare through AI is thus often also linked to other values, such as empowerment, participation, equity and access to healthcare.

### 3.2 Artificial intelligence by the people: Democratizing artificial intelligence in medicine and healthcare by multiplying developers, evaluators, and users

The second vision of democratization of AI refers to facilitated access to AI-technologies in terms of design and/or use. Democratizing AI in this respect means making machine learning accessible to non-domain specialists (Dibia et al., 2018; Traub et al., 2019; Gupta, 2020; Kobayashi et al., 2019; Mulvenna et al., 2021; Vanhorn and Çobanoğlu, 2021). The aim is to enable those without technical expertise on AI, such as healthcare professionals as well as biomedical researchers, to handle AI-technologies. Some authors describe this democratization of AI as an already ongoing process, which will contribute to a widespread use of AI in biomedical research and healthcare practices. According to this view, high-performance computer hardware, cloud machine learning tools, accessible software, and affordable online education have already democratized the creation and use of AI (Dibia et al., 2018; Bond et al., 2019b; Mulvenna et al., 2021; Saldívar-González et al., 2022). “Democratizing”, in this understanding, is mostly discussed regarding better access to knowledge and tools. Some authors note that health professionals lack the required knowledge and skills for handling AI, which also negatively influences their attitudes towards the technologies (Allen et al., 2019). A basic knowledge of how algorithms work, what their limits are, and how to evaluate them for clinical practice is thus needed.

An important aspect in the context of facilitating better access to AI technologies is the often-lacking infrastructure in hospitals and other healthcare facilities for engaging with AI development (Allen et al., 2019). One approach to overcome this barrier is to provide toolkits or other ready-made solutions for developing and applying AI (Dibia et al., 2018; Sikpa et al., 2019; Vanhorn and Çobanoğlu, 2021). Vanhorn and Çobanoğlu (2021) suggest a virtual reality (VR)-platform as a simplified environment in which users can design, train, and evaluate models. Instead of coding, users handle data sets, in this case images, in an immersive environment where they can grab data sets and shift or sort them. This immersive experience is meant to enable a more intuitive model development without any coding skills. Another approach is the provision of platforms for code-free automated machine learning (AutoML) interfaces, which is explicitly framed as an empowerment of healthcare professionals and biomedical researchers (Nature Machine, 2021).

Thus, the second cluster of visions of democratizing AI shares the technological optimism with the first vision yet problematizes the identity of the visioners of AI. In this vision AI shifts from a transformative agent that renders medicine and healthcare more democratic to an object *in* medicine and healthcare that needs to be rendered more democratic—or indeed, to be democratized. Here, democratizing AI refers to making tools to render AI accessible to biomedical professionals. In this vision, the demos refers to biomedical professionals, who ought to be involved in the development of AI or be able to use models developed with the help of AI. In turn, democratizing AI in this way helps to empower biomedical professionals, augment their expertise, while also disseminating the use of AI in biomedicine. It is important to note that especially Big Tech companies like Amazon, Google, and Microsoft are key players in this vision of “democratizing” AI (Nature Machine, 2021).

### 3.3 People in artificial intelligence: Democratizing access to and oversight of data

The third cluster of visions shares the second’s understanding that the development of AI-based systems needs to be democratized, focusing on access to and oversight of data used to develop AI-based technologies. It addresses the issue that algorithms are often developed, trained, or validated on data from a single institution (Allen et al., 2019; Traub et al., 2019; Gupta, 2020), which is one of several sources of data bias. We identified two related visions on how access to, and oversight of, data can be democratized: strategies for distributive learning and the establishment of ‘data commons’ or “data trustees”.

One vision suggests various technical approaches to “democratize” the data used to develop AI, by making data more representative. Some authors suggest approaches for distributed training of AI models with decentralized data like federated, distributed, or split learning for facilitating data sharing (Allen et al., 2019; Lyu et al., 2020). Another strategy is transfer learning (TL), where instead of training new models at each hospital, pretrained models can be used and adapted, which reduces the number of data sets needed (Gupta, 2020). Other approaches focus on enabling sharing between institutions. For instance, Traub et al. developed a data ecosystem that serves as infrastructure for sharing assets such as data, algorithms, ML models, systems, services, and compute resources (Traub et al., 2019). Used as a marketplace, this ecosystem could facilitate easier access to these assets.

Another vision is centered around strategies for making data more accessible. Democratizing AI in this respect means to facilitate open access to data sets (Bhattacharya et al., 2021). Bhattacharya et al. identify three crucial factors of democratizing health data: Discoverability in data repositories through the provision of meta data, accessibility of data using websites, tools, and interfaces, and interoperability through standardization of data sets (Bhattacharya et al., 2021).

Thus, the third vision of democratizing AI builds on the second vision’s understanding that AI in medicine and healthcare needs to be democratized to unleash its transformative potential and problematizes the “means of production” of AI. It does not focus on the hard- and software, or on the skills needed to use them in practice (as the second vision), but addresses the nature of the data needed to develop, train, and evaluate AI. This vision underlines that there is a shortage of high-quality data in biomedicine and healthcare, that can represent the variety of patients, groups, and people that ought to benefit from AI-based technologies in medicine and healthcare. While this vision overlaps with the second vision as being primarily about enabling access to the means of production of AI, here the demos does not only relate to the users of data (i.e., biomedical professionals) but also to the contributors of data or the people whose traces are in the data.

### 3.4 Making artificial intelligence an object of the rule by the people

A fourth visions of democratizing AI calls to transform AI into an object of the rule by the people, suggesting that “AI should be subject to novel or different forms of democratic governance” (Himmelreich, 2022, p.3). Just as AI has the potential to contribute to human wellbeing, it can also be used, or abused, to achieve undesirable ends. Thus, there is a sense that the development and adoption of AI-based systems cannot be left in the hands of developers, technicians, or big tech alone (Garvey, 2019). To ensure that AI will “serve the public good” (Nemitz, 2018, p.7), AI and the professionals that develop and use it need direction, oversight, and democratic governance by the people, or by authorities acting on behalf of them.

We identified two versions within this cluster of visions of democratic governance for medical AI, which draw upon different approaches towards democratic governance. One vision builds upon traditions of direct democratic governance and calls for the involvement or engagement of people affected by AI in the development and oversight of AI—or public participation for short. This vision builds upon practices of public participation and public engagement, which have become salient in the governance of emerging technologies over the past decades. While they have taken shape in very different ways, participatory practices are sustained by the understanding that people directly or indirectly affected by emerging technologies (as consumers, patients, or citizens), should have a say on the development, use, or oversight of said technologies (Hagendijk and Irwin, 2006; Felt and Fochler, 2008; Gottweis et al., 2008; Doubleday and Wynne, 2011; Braun and Könninger, 2018). In the case of AI, public participation is also referred to as “co-creation” or “co-design (Donia and Shaw, 2021). Involving the public in the development and oversight of AI-based systems is expected to render the latter socially robust and acceptable. Calls to include publics in the design of AI-based systems are tied to the normative understanding that people affected by AI should be involved in their development, or—drawing on the term Harambam and colleagues used for algorithmic news recommenders—people should have “voice” (Harambam et al., 2018) if they are affected by it. Moreover, calls for including publics in the design of AI are also tied to the normative expectation that people’s practical knowledge can render AI more intelligent, such as by helping to identify needs (Barclay, 2020), or by learning from publics how they define ethical values (Wong, 2020).

We can also find such calls to engage publics in documents of international and professional societies. For instance, a guidance from the World Health Organization (WHO) on the “Ethics and governance of artificial intelligence for health” suggests that AI technologies should both be “designed by and evaluated with the active participation of those who are required to use the system or will be affected by it, including providers and patients” (World Health Organisation, 2021, p.29) to ensure “inclusiveness and equity”. Similarly, the United Kingdom Academy of Medical Sciences deemed “ongoing engagement with patients, the public and healthcare professionals (…) critical to ensuring new AI technologies respond to clinical unmet need, are fit for purpose, and are successfully deployed, adopted, and used.” (Academy of Medical Sciences, 2019).

Another version of the vision of democratizing AI by rendering it an object of democratic governance builds upon theories of representative democracy, by entrusting authorities with overseeing the development and use of AI on behalf of the people. While this vision shares a common ground with calls for public participation, calls for democratic governance through enforceable regulations are also responsive to the proliferation of ethical principles for AI over the past few years, which have been developed with the involvement of Big Tech (Mittelstadt, 2019). While these principles are welcomed, voluntary compliance is often deemed insufficient to ensure that AI will help to enable desirable futures. For instance, Paul Nemitz (Nemitz, 2018, p.1) argues that the “key question for AI in democracy” is what can be left to “ethics”, or the voluntary self-governance of the industry along a set of ethical principles and practices, “and which challenges of AI need to be addressed by rules which are enforceable and encompass the legitimacy of democratic process, thus laws.”

This vision on democratic AI governance allocates responsibilities to state actors and regulatory authorities to make AI a “subject to the rules set by democracy in law” (Nemitz, 2018, p.10). Over the past few years, several state authorities and international and supranational organizations have begun to address how to govern AI through law and law-like measures. For instance, the European Commission (EC) has begun to elaborate on an approach that “places people at the centre of the development of AI (human centric AI) and encourages the use of this powerful technology to help solve the world’s biggest challenges” (Ulnicane, 2022, p.261). In a White paper on AI published in February 2020, EC outlined a risk-based common European regulatory framework to ensure “that new technologies are at the service of all Europeans—improving their lives while respecting their rights.” (European Commission, 2020, p.1). A central aim of the EC is to oversee AI in such a way as to allow “people [to] to be able to trust it.” (European Commission, 2020, p.1) page 1.

Thus, a fourth narrative on democratizing AI builds upon the first three meanings, while also extending the scope of democratizing AI. While it capitalizes on the narrative of AI as a powerful transformative technology, it draws on practices and theories of participatory and representative democracy to underline that AI needs to be made an object of the rule by the people to ensure that it will be developed and used for the people and accepted and trusted by them. In this vision, the demos refers to the people directly or indirectly affected by AI and thus to the public, who should either be engaged in the development of AI or be represented by authorities who govern AI on behalf of them.

## 4 Discussion

We have mapped different ways the term “democratizing AI” is used in the discourse on AI in medicine and healthcare, describing four clusters of visions that we have distilled from the material. In the following, we discuss our findings along the object of democratization, the demos, and the type of democracy tied to each of the four cluster of visions of democratization. We contextualize the results with research on medical ethics and ethics of digital health technologies.

### 4.1 The object of democratization: The problem to be solved

The object of democratization, i.e., the practices or structures to be democratized, varies between the four visions we identified. The first vision discusses the democratization of healthcare through AI. The other three visions focus on the democratization of AI in medicine and healthcare, articulating different visions of which dimensions of AI ought to be democratized and of who ought to be engaged. The first vision defines medicine and healthcare as the object of democratization. According to this vision, paternalistic and dysfunctional healthcare systems should be transformed into consumer-oriented non-hierarchical health markets. A more personalized and autonomy-empowering healthcare system, especially in HICs, and better access to healthcare, especially in LMICs, could be the main benefits of the democratization of medicine and healthcare through AI. The second and third cluster of visions identify AI-based technologies as the object of democratization. Enabling better access to health data, models, and algorithms is the main objective in this context. The free exchange of data, knowledge, and technologies is key to improving the quality of AI-based technologies and enabling healthcare professionals to contribute to development and usage of these technologies. In the fourth vision on democratization, the practices of people who envision, develop, and use AI are deemed to be in need of being made an object of democratic governance.

A tendency towards technical solutionism cuts across all four visions, i.e., the notion that genuinely social or political problems can be fixed through technological innovations or the applications of technology (Morozov, 2013; Howard, 2021), even if this solutionism is stronger in the first vision and weaker in the fourth vision. An example is Topol’s concept of deep medicine, where AI offers “a technological solution to the profound human disconnection that exists today in healthcare” (Topol, 2019, p.272). In some visions, the solutionist approach is rather vague, simply framing democratization of healthcare as a good that medical AI can deliver (Weissglass, 2021). In others, explicit problems and their AI-based solutions are identified. For example, AI may fix a crucial problem of the supposedly dysfunctional healthcare system, missing care, because technology “doesn’t complain, can work, doesn’t get tired” (Topol, 2019).

### 4.2 The demos

We identified the demos tied to each of the four visions of democratization we derived from our material. In the first vision, democratization of healthcare through AI, the demos relates to individuals and groups empowered by AI. In the second vision of democratizing access to tools to develop AI, the demos consists primarily of biomedical professionals (including researchers and clinicians) who lack technical expertise on AI. In the third vision of democratizing data, the demos is more ambiguous, referring to biomedical professionals who ought to have access to data and to groups of patients who ought to be represented in data. The demos of the fourth vision is closest to the demos as we (and political theory) know it. It refers to the people who would be directly and indirectly affected by AI or to the “public” (Dewey, 1927). They are deemed to be entitled to have a say on how, by whom, and for which purposes AI-based technologies are developed and used—either by having a direct say on them in participatory settings, or by being represented by authorities who govern AI on behalf of publics. Thus, the way in which the demos is tacitly defined and normatively framed in the four visions differ. The difference is particularly visible when the demos of the first vision is compared with the demos of the fourth vision.

The prevalent framing of individuals in the first vision of democratization is the patient-as-consumer, that is, typical for digital health (Lupton, 2013). Two enabling factors of empowerment are especially relevant in this regard: data-ownership and mobile health (mHealth) technologies. For Topol (Topol, 2019), the empowered patient is a consumer, defined by ownership of their own health data. He gives a list of reasons, of which the first two are “It is your body. You paid for it.” (Topol, 2019, p.264). Ownership of their own health data empowers patients to act as consumers in the medical encounter. The underlying assumption is that as data-owners and consumers, patients are in a stronger position vis-à-vis medical professionals. Democratization is framed as the antagonist to medical paternalism. The empowered patient is a consumer of health services and an owner of data that emancipates themselves from a paternalistic system.

However, the basic narrative of empowerment through engagement with one’s own health data and self-management practices is highly problematic. The idea that AI-based technologies and especially mHealth solutions enable empowerment of patients and lead to more autonomy has been widely criticized (Lupton, 2013; Sharon, 2016; Rubeis et al., 2018; Morley and Floridi, 2020; Rubeis, 2020). Morley and Floridi state that there is simply no clear evidence for the claim that mHealth technologies strengthen patient autonomy (Morley and Floridi, 2020). Furthermore, it has been shown that the supposed empowerment in digital technologies is often a fig leaf for hidden agendas (Rubeis et al., 2018). The rhetoric of autonomy and empowerment is often used to sugarcoat commodification and work optimization using AI-technologies within the healthcare system (Dillard-Wright, 2019). Following Lupton (Lupton, 2013), the emphasis on patient engagement and the patient-as-consumer approach is the outcome of a “neoliberal” agenda that promotes the shift of responsibilities from the collective to the individual. In a similar vein, Sharon (Sharon, 2016) interprets activation of patients as a means of cost-reduction in the health sector, e.g., by reducing contact with health professionals.

In the fourth vision, individuals are not framed as consumers, but as members of a public consisting of citizens with rights (European Commission, 2020; World Health Organisation, 2021). This vision focusses on public engagement and co-design in AI-development as well as regulation and governance. The inherent tension between this vision and the economic and political reality is obvious (Wilson, 2022). AI is almost exclusively shaped by the private sector, which also influences the development of standards and regulations, and public citizen participation in development and decision-making process concerning AI is virtually non-existent.

### 4.3 The democracy: A libertarian utopia

Building on the dominant patient-as-consumer approach regarding the demos, it is not hard to make the connection to the corresponding type of democracy that underpins the first vision of democratization. By taking the ideas of self-ownership and individual responsibilities as a given, the first vision of democratization of healthcare through AI, channels Lockean individualism typical for libertarian thinking (Olsthoorn, 2019). It reduces democracy to the libertarian idea of being free from external interference by authorities, in our case- medical paternalism. The connection to libertarian theories is sometimes implicit, as in the case of Topol’s approach, sometimes explicit, e.g., by referring to libertarian authors (Montes and Goertzel, 2019). Two ideas are crucial for this libertarian approach: engagement and self-management on behalf of users and decentralized access to data.

Engagement and self-management manifest themselves in the aforementioned strong focus on AI-based mHealth technologies. We have seen that these technologies are promoted as enablers of patient empowerment. The use of mHealth for data collection is also sometimes framed as a means to circumvent economic, legal, or political restraints to improve healthcare services (Weissglass, 2021). When faced with supposedly dysfunctional structures and infrastructures, patients should take their health into their own hands. Healthcare is thus framed as an individual responsibility.

Calls for free-flowing data and universal access to technologies also fit well with typical libertarian ideas. Decentralized data repositories and block chain solutions are promoted as pillars of a more democratic healthcare (Montes and Goertzel, 2019; Traub et al., 2019; Bhattacharya et al., 2021). Following this notion, absence of restrictions and access to data and technologies have a democratizing effect.

However, the involvement of Big Tech in medicine and the immense power, that is, given to a rather small number of companies by letting them handle large amounts of data could also be seen as a direct threat to democracy instead of enabling a more democratic healthcare. The idea that a data-based free-market utopia will make healthcare more democratic suffers from a specific libertarian blind spot, i.e., the awareness for structural inequalities and asymmetric power and property relations. These issues manifest themselves in the so-called big data divide between those who provide data and those who possess the means to process data (Mittelstadt and Floridi, 2016). Granting broader access and enabling restriction free data-sharing alone will not resolve this issue, since the intellectual (knowledge) and material resources for processing data remain unequally distributed. The focus on free-flowing data and access ignores the existing digital power concentration that shapes the infrastructures and required markets (Nemitz, 2018).

This begs the question of democratic control, which is a crucial aspect when it comes to big data in healthcare (Gould, 2019; Sangiovanni, 2019). In this regard, democratic control has three main objectives: ensuring that all that are affected by decisions or actions can participate in the decision-making process (all-affected principle), focusing on the common good, and enabling individuals to make use of their freedom. Democratic control thus requires participation, deliberation, and representation that aim at compensating for or preventing unequal power and property relations (Gould, 2019). In order to democratize AI-based healthcare technologies, the infrastructure for developing and distributing them would have to be an object of democratic control instead of trusting in an invisible hand.

### 4.4 Strengths and limitations

To the best of our knowledge, this is the first study that explored in depth, the different versions of terms “democratization” and “democratizing” that are used in the context of AI in medicine and healthcare. Qualitative methods allowed us to explore and map the emerging topic in detail.

Our mapping of the visions also has limitations, which need to be considered to qualify our results. First, the use of peer-reviewed articles and documents in English limited our attention to comparatively privileged voices in the discourse on AI in medicine and healthcare. A more thorough mapping of visions of democratization, which could capture alternative visions of democratizing AI, would need to extend the materials to other languages and materials. Second, in light of the emerging nature of discourses on democratizing AI in healthcare and medicine, we have neither quantified our results, nor explored the relationship between visions articulated in documents to practices, institutions, and materialities outside the documents. We also did not analyze the political, social, and economic forces that shape the clusters we identified in detail. Further research is needed to address this topic. We see our paper as the first step in that direction.

## 5 Conclusion

In this paper, we mapped the different ways the terms “democratization” and “democratizing” are used in the discourse on AI in medicine and healthcare and performed a normative analysis of the findings, embedding them in normative engagements with data-intensive technologies. We derived four clusters of visions of democratizing AI from our qualitative analysis of peer-reviewed articles and grey literature: A first cluster of visions focusses on AI for the people and aims at democratizing medicine and healthcare through the further implementation of AI. These visions frame AI as a technological fix to problems in healthcare systems, such as inequity and access barriers, and lead to a personalization of medical practice. A second set of visions shifts to AI by the people and encompasses visions of democratizing AI in medicine and healthcare by facilitating better access to AI technologies for healthcare professionals without a background in data science or informatics. According to this vision, the provision of knowledge and tools, e.g., ready-made toolkits and code-free interfaces, may facilitate better access to AI technologies and enable medical practitioners to contribute to developing AI-based systems and better integrate them in their practice. Access also plays a crucial role in the third set of visions that we described as people in AI. They focus on democratizing access to and oversight of data. The aim is to make the data, that are used to develop, train, and evaluate AI, more representative as well as accessible by applying various strategies for decentralizing data generation and broader dissemination of data. A fourth cluster of visions seeks to make AI an object of the rule by the people and thus a matter of democratic governance. Democratic governance may imply participation of publics in the design and development processes of AI-based systems or the regulation of AI by democratically legitimized authorities.

Our normative analysis shows that democratization in the context of medical AI can be seen as an example of the kind of rhetoric Daub described that aims at shaping our view of how we could or should live. Weak and strong versions of technical solutionism cut across all visions. The supposed potential of AI technologies to fix primarily social and political problems not only raises false hopes but may also obscure the need for alternative solutions. We also highlight that the envisioned democratization in most visions mainly focuses on patients as consumers and relies on or limits itself to free market-solutions. This rather libertarian understanding of democracy ignores the need for formulating rights that ensure fair distribution and democratic control of AI and the services it provides. This is especially an issue since the development and implementation of AI is largely driven by a small number of companies usually referred to as Big Tech. Protecting the interests of those affected by AI and facilitating a real democratization *of* AI in medicine and healthcare, or even democratization of medicine and healthcare through AI thus requires a rights-based approach instead of technological solutionism or reliance on market forces.

However, the different ways in which the term democratization is used also suggests that this vision is not universally shared. While the imagination that AI-based technologies will help us fix problems in medicine and healthcare underpins all these visions, different understandings of the identity and the place of the demos also show that—who should have the power to envision these futures and who ought to be involved in striving for them, is contested.

This does not mean that democratization is a false term in this context. Our mapping of the ways in which this term is used has helped us to show that it helps to raise important questions about the development and use of AI in medicine and healthcare, about the kind of futures that we strive to attain through AI-based technologies, and who we think ought to be involved and have a say when working towards that future. Specifically, it directs attention to implicit definitions of the demos that ought to be engaged in the development, use, and oversight of AI, and different practices of engagement. In the current uses of the term, these questions are often answered tacitly. The nature of the demos and its appropriate place are presumed. Developing “democratizing AI” into a more robust concept could help us think more systematically through the implicit normativity in the development of AI-based systems. It could be used as what Herbert Blumer (Blumer, 1954) named a “sensitizing concept”—i.e., a concept that makes us attentive to questions to be asked and issues to be taken care of.

For democratization and democracy to be more than misnomers here, a much more substantial theoretical foundation is needed. Democratization in the context of AI in healthcare requires defining and envisioning a set of social goods. It also needs deliberative processes and modes of participation to ensure that those affected by AI in healthcare have a say on its development and use.

## Data Availability

The original contributions presented in the study are included in the article/supplementary material, further inquiries can be directed to the corresponding author.
